# An unusual case of severe asphyxia with the fetal position unexpectedly inverted in a malformed uterus: a case report

**DOI:** 10.1186/s13256-024-04524-0

**Published:** 2024-04-26

**Authors:** Jiro Abe, Takashi Nasu, Ayumu Noro, Junko Tsubaki

**Affiliations:** 1 Department of Pediatrics, JCHO Hokkaido Hospital, 3-18, Nakanoshima 1 Jyou 8 Tyoume, Sapporo, Japan; 2 Department of Pediatrics, Graduate School of Medicine, Hokkaido University, Kita-15, Nishi 7, Kita-Ku, Sapporo, 060-8638 Japan; 3 Mitochondrial Redox Biology, Medical Research Council Mitochondrial Biology Unit and Department of Medicine, University of Cambridge, The Keith Peters Building, Cambridge Biomedical Campus Hills Road, Cambridge, CB2 0XY UK

**Keywords:** Inverted fetal position, Neonatal asphyxia, Hypoxic-ischemic encephalopathy, Late preterm, Uterine anomalies, Tocolysis

## Abstract

**Background:**

We present a severe neonatal consequence due to the unexpected and crucial inversion of the fetal position after sudden termination of tocolysis during early labor of a woman with congenital uterine anomaly. It has been reported that congenital uterine anomalies latently affect the fetal position. The clinical pitfalls in childbirth with uterine anomalies are discussed here on the basis of clinical evidence.

**Case presentation:**

At a perinatal medical center in Japan, a 29-year-old Japanese mother who had a history of bicornuate uterus, received tocolysis to prolong her pregnancy for 5 days during the late preterm period after preterm-premature rupture of the membrane. She gave birth to a 2304 g male neonate of the gestational age of 35 weeks and 5 days with severe asphyxia by means of crash cesarean section for fetal sustained bradycardia after sudden termination of tocolysis. We found the fetal position to reverse from cephalic to breech position during early labor. He ended up having severe cerebral palsy after brain cooling against hypoxic-ischemic encephalopathy for 3 days. The mechanism of inversion from cephalic to breech position without amnionic fluid remains unclear, although women with a known diagnosis of a uterine anomaly have higher risk of adverse outcomes such as malpresentation.

**Conclusions:**

When considering the clinical course of this case on the basis of the medical reports, we suspected that uterine anomalies and changes in intrauterine pressure could cause fetal malpresentation and adverse neonatal outcomes.

## Background

Uterine abnormalities may be overlooked in women with successful reproductive outcomes, but one study estimated that, even in women with normal pregnancy outcomes, the incidence of congenital uterine anomalies is approximately 3%. The likelihood of fetal malpresentation at the time of delivery is notably increased by the presence of uterine anomalies [1]. A case is presented where a mother with bicornuate uterus received tocolytic treatment with β-stimulants after surpassing 35 weeks of gestation. Following the discontinuation of tocolysis associated with the onset of labor, the fetus experienced distress and malpresentation, ultimately resulting in severe cerebral palsy in the child. There are no existing case reports that show a change in fetal presentation during labor with uterine anomalies resulting in fetal asphyxia.

## Case presentation

**Fig. 1:**
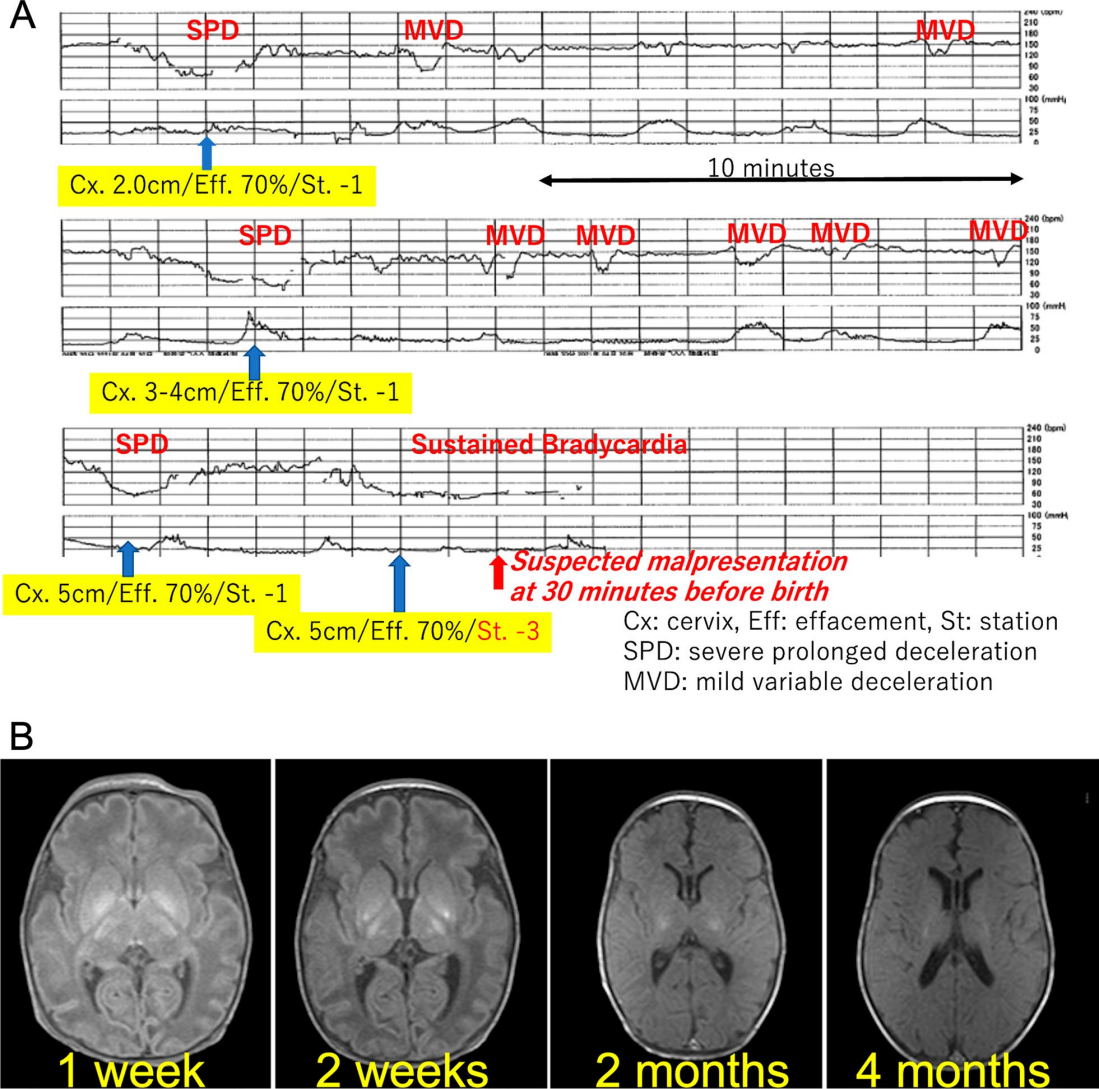
(A) Cardiotocography immediately before birth. Non-reassuring patterns repetitively emerged 1 hour prior to birth. Pelvic examinations revealed a fixed cephalic position. At half an hour, fetal bradycardia was sustained while the pelvic examination indicated that the fetal head was unexpectedly floating. (B) Sequential brain magnetic resonance imaging (MRI). According to sequential brain MRI findings, his lesions post hypoxic-ischemic encephalopathy (HIE) were mainly located in the basal ganglia and the brain stem

A 29-year-old Japanese mother who had a history of bicornuate uterus gave birth to a 2304 g male neonate of the gestational age of 35 weeks and 5 days with severe asphyxia. She was a primigravida without health issues, and her pregnancy course, including changes in maternal body mass index (BMI) and gestational weight gain, was smooth. She was admitted to our hospital to receive tocolysis treatments using ritodrine hydrochloride because of preterm premature rupture of membrane at 35 weeks’ gestation, receiving antibiotics, no antenatal corticosteroids, and no magnesium sulfate. The ultrasound examinations revealed overall fetal growth, reduced amniotic fluid, and a fetal vertex position. A total of 3 hours before the birth, the administration of the tocolytic agent, by ritodrine hydrochloride using the maximum dose of 200 µg per minute, was terminated to promote vaginal delivery, and she was transferred to a delivery room. Non-reassuring patterns repetitively emerged at 1 hour prior to the birth, which were only confirmed by cardiotocography without an ultrasound examination performed at that time (Fig. 1A). General fetal resuscitation such as maternal oxygen administration and intravenous infusion of a liter of non-glucose crystalloid without acute tocolysis was used as part of the obstetric management of labor, while preparing for cesarean delivery for fetal distress. Quick pelvic examinations ensured the fetus’s cephalic position while observing the mother in preparation for an emergency cesarean operation, although the fetal position during delivery was not confirmed by ultrasound. Half an hour before the birth, fetal bradycardia was sustained while the pelvic examination indicated that the fetal head was unexpectedly floating (Fig. 1A). A crash cesarean section was performed, where he was found to be in a breech presentation. We found quite little amniotic fluid without the evidence of meconium-stained amniotic fluid or cord coiling. The pathological findings of the placenta and umbilical cord proved only mild chorioamnionitis without any evidence of delivery injury or anomaly afterward.

After delivery he presented with bradycardia and deep cyanosis without breathing, muscle movements, and reflections. Because his asphyxia turned out to be refractory to routine resuscitation, he was intubated after 1 minute. His skin color rapidly became pink, and the heart rate returned to a normal range without the recovery of muscle movements and reflex actions. He received an appearance, pulse, grimace, activity, and respiration (APGAR) score of 1 at 1 minute and 3 at 5 minutes; the arterial cord blood sample was not available, because of technical difficulty in sampling umbilical cord blood. He needed special care that included mechanical ventilation and correction of mixed acidosis (pH 6.85, pvCO2 77 mmHg, HCO_3_^−^ 12.6 mmol/l at 15 minutes after birth), and then he was given phenobarbital. At 1.5 hours after birth, he was transferred to another tertiary care hospital where he received therapeutic hypothermia for hypoxic-ischemic encephalopathy; the Sarnat grade was moderate, and the Thompson score was calculated as 16 points [2]. He was a late preterm and low-birth-weight newborn with no congenital anomalies or other problems that would be predictive of neonatal asphyxia through newborn screening especially focusing on the brain, heart, or metabolism. We could not find clinical and pathological evidence of his severe asphyxia in the end. Chromosomal testing was not conducted. He ended up having severe cerebral palsy after brain cooling for 3 days. His sequential brain MRI findings supported the severity of the encephalopathy that mainly affected the basal ganglia and brain stem (Fig. 1B). He is now 9 months of age and remains in bed with special healthcare requirements that include tube feeding, while presenting with dystonia with severe mental developmental retardation.

## Discussion and conclusions

The mother had some delivery risks as follows: a uterine anomaly, absent amniotic fluid after preterm-premature rupture of membrane, and threatened late-preterm labor. The placental blood flow in mothers with congenital uterine anomalies is reduced, and there is a predicted decrease in the reserve capacity for blood supply to the fetus, particularly during delivery. When a mother has congenital uterine anomalies, there is a 5-fold increased risk of preterm birth and a 20-fold increased risk of placental abruption [3]. This case is believed to be caused by circulatory insufficiency between the mother and fetus, with the influence of congenital uterine anomaly likely playing a background role. Increased intrauterine pressure might have occurred by abrupt termination of tocolysis with the lack of amniotic fluid, which would make the fetal status worse, although there was no evidence of excessively rapid uterine contractions in the tocography (Fig. 1A).

Uterine anomalies are known to significantly elevate the chances of fetal malpresentation during delivery. According to the meta-analysis by Chan, the likelihood of fetal malpresentation was found to be higher in cases of arcuate uterus, unification defects, and canalization defects, with the odds being 2.53 [95% confidence interval (CI) 1.54–4.18; *p* < 0.001], 3.87 (95% CI 2.42–6.18; *p* < 0.001), and 6.24 (95% CI 4.05–9.62; *p* < 0.001) times, respectively [4]. Furthermore, a retrospective study by Hua and colleagues, which encompassed all types of uterine anomalies (including uterine septum, unicornuate uterus, bicornuate uterus, and uterine didelphys), revealed that women with these anomalies were 8.6 times more likely to experience breech presentation of the fetus compared with women with standard uterine anatomy (95% CI 6.2–12.0; *p* < 0.01) [5]. Additionally, a comprehensive retrospective cross-sectional study, examining a total of 109,736 singleton infants (both preterm and full-term), of which 4535 were breech at birth, determined that women with any form of uterine malformation had an almost 10-fold increase in the likelihood of breech fetal presentation (odds ratio, 9.47; 95% CI 6.77–13.25) [6]. Possible causes are thought to be changes in intrauterine and external pressure, for example, the effects of uterine malformations, the sudden discontinuation of uterine contraction inhibiting drugs, and the transfer from the delivery table to the bed. The unexpected inversion of the fetal position with very little amniotic fluid during early labor would have led to the poor consequence, causing the umbilical cord to twist and consequently leading to the interruption of placental blood flow.

It may have been unavoidable, but we can suggest two preventive plans for this case. One plan would be ongoing expectant management with or without tocolysis. The issue of whether to suppress or allow progressive labor to proceed during the late-preterm period remains controversial [7]. If waiting for labor while inhibiting uterine contractions, it is necessary to carefully monitor changes in intrauterine pressure when stopping tocolytic agents. The other one would be planned earlier delivery including elective cesarean operation. Bicornuate uterus has been reported to be a risk factor for unsuccessful vaginal delivery [8]. A major meta-analysis discovered that the likelihood of undergoing a primary cesarean delivery was 2.6 times higher for women with congenital uterine anomalies (adjusted odds ratio [aOR], 2.6; 95% CI 1.7–4.0; *p* < 0.01) [5]. Additionally, a retrospective cohort study over a decade at a French university hospital assessed women known to have uterine malformations, focusing on the baby’s presentation and the method of delivery. In this group, women with uterine abnormalities showed a significantly increased incidence of breech presentations (36.51% as opposed to 4.52%; *p* < 0.01) and cesarean deliveries (55.26% compared with 18.70%; *p* < 0.01), in contrast to women with normally formed uteri [9].

Women with congenital uterine anomalies face significantly higher risks of preterm birth, placental abruption, fetal malpresentation, and breech presentation. Several studies highlight the increased odds of complications such as fetal malpresentation and breech births, indicating a need for careful monitoring and possibly alternative delivery plans, including elective cesarean operations. Our report concludes with suggestions for managing such high-risk cases, emphasizing the importance of careful monitoring or possibly opting for an earlier planned delivery to mitigate risks.

## Data Availability

The approval from the parent allowed us to use the patient’s data and report this case with data anonymization.
